# Supplementary material for: Work-Related Traumatic Stress Response in Nurses Employed in COVID-19 Settings
Source: Int J Environ Res Public Health. 2022 Sep 3;19(17):11049. doi: 10.3390/ijerph191711049 (PMC9518142; doi:10.3390/ijerph191711049)
Supplement: Supplementary file 1 [file ijerph-19-11049-s001.zip › ijerph-1882315-supplementary.pdf]

SUPPLEMENTARY TABLES

Supplementary Table S1 (Table S1). Presentation of differences in mean scores of the main variables and groups of socio-demographic, education, and employment variables (N=233)								
			t-test for Equality of Means					
							95% Confidence Interval of the Difference	
		Mean (SD)	t	df	Sig. (2-tailed)	Std. Error Difference	Lower	Upper
**GENDER**								
**Distress from being avoided due to working in a Covid setting**	Male	6.3 (2.7)	-2.798	231	.006	.36091	-1.72082	-.29864
	Female	7.3(2.6)						
**POST-GRADUATE EDUCATION**								
**Satisfaction from Covid-related guidance provided by administrators**	No	4.6(2.9)	-1.920	231	.056	.4320	-1.6806	.0216
	Yes	5.4(2.8)						
**Satisfaction from the provided PPE**	No	5.8(2.3)	-2.498	231	.013	.34054	-1.52171	-.17978
	Yes	6.6(2.0)						
**NIGHT SHIFTS**								
**Satisfaction from Covid-related guidance provided by administrators**	Less than 5	5.6(2.7)	4.299	230	.000	.3693	.8601	2.3154
	More than 5	4.0(2.8)						
**Satisfaction from the provided PPE**	Less than 5	6.6(2.0)	3.841	230	.000	.29506	.55205	1.71478
	More than 5	5.5(2.4)						
**DEATHS PER DAY BY ANY CAUSE**								
**Emotional exhaustion**	Less than 5	7.23(2.4)	-1.970	231	.050	.502	-1.978	.000
	More than 5	8.22(1.3)						
**Satisfaction from the provided PPE**	Less than 5	6.2(2.2)	3.866	231	.000	.49295	.93455	2.87704
	More than 5	4.3(2.8)						
**RANKING**								
**Emotional exhaustion**	Staff Nurse	7.26(2.3)	-2.653	19.415	.016	.406	-1.925	-.228

	Head nurse/ under head nurse	8.33(1.5)						
**Satisfaction from the quality of provided care**	Staff Nurse	6.8(2.0)	-2.413	231	.017	.5409	-2.3709	-.2395
	Head nurse/ under head nurse	8.1(1.8)						
**Distress from being avoided due to working in a Covid setting**	Staff Nurse	6.9 (2.7)	-2.234	231	.026	.71387	-3.00164	-.18857
	Head nurse/ under head nurse	8.5(1.7)						
			**ONE WAY ANOVA**					
		**Mean (SD)**	**Sum of Squares**	**df**	**Mean Square**	**F**	**Sig.**	**Post-hoc test (Sceffe) p value**
**TOTAL WORK SATISFACTION**			36.106	2	18.053	3.801	.024	(1)-(3) 0.027
**Work city**	Famagusta [reference hospital] (1)	7.00(1.7)						
	Limassol(2)	6.06 (2.0)						
	Nicosia(3)	5.94(2.4)						
**SATISFACTION FROM COVID-RELATED GUIDANCE PROVIDED BY ADMINISTRATORS**			320.183	2	160.091	22.293	.000	(1)-(3) p<0.001 (1)-(2) p<0.001
**Work city**	Famagusta reference hospital (1)	7.14(1.8)						
	Limassol (2)	4.82(2.4)						
	Nicosia (3)	3.94 (3.0)						
**SATISFACTION FROM PROVIDED PPE**			114.607	2	57.304	11.723	.000	(1)-(3) p<0.001
**Work city**	Famagusta reference hospital (1)	7.04(1.8)						
	Limassol (2)	6.46(1.8)						
	Nicosia (3)	5.33(2.5)						
**SATISFACTION FROM COVID-RELATED GUIDANCE PROVIDED BY ADMINISTRATORS**								

**Age**	Over 45 years (1)	5.81(2.7)	73.169	2	36.585	4.445	.013	(1)-(3) 0.026
	35-44 years (2)	5.20(3.0)						
	25-34 years (3)	4.36(2.8)						
**SATISFACTION FROM PROVIDED PPE**								
**Age**	Over 45 years (1)	7.08(2.3)	66.578	2	33.289	6.615	.002	(1)-(3) 0.002
	35-44 years (2)	6.23(1.9)						
	25-34 years (3)	5.61(2.3)						
**SATISFACTION FROM PROVIDED PPE**			64.046	2	32.023	6.269	.002	(2)- (1) 0.003
**Number of offspring**	None (1)	5.23(2.5)						
	1-3 children (2)	6.33(2.0)						
	More than 3 children (3)	6.50(2.5)						

Supplementary Table S2 (Table S2). Differences between severity of work-related traumatic stress response (STSS-M total score) and demographic characteristics (age, gender) (t-test) (N= 233)

	Gender (p=0.004)			
**Severity of symptoms (STSS-M total score)**	Male		Female	
No or Low intensity	12.8%		5.4%	
Mild intensity	24.4%		17.7%	
Moderate intensity	14.0%		17.0%	
High intensity	19.8%		9.5%	
Severe intensity	29.1%		50.3%	
	Age (p=0.016)			
Severity of symptoms	**<25 years**	**25-34 year**	**35-45 years**	**<45 years**
No or little intensity	-	84.2%	5.3%	10.5%
Mild intensity	-	53.2 %	36.2%	10.6 %

Moderate intensity	-	51.4	37.8	8.1
High intensity	2.7%	48.4	19.4	32.3
Severe intensity	-	61.5	21.9	16.7
